# Knowledge atlas and emerging trends on ncRNAs of osteosarcoma: A bibliometric analysis

**DOI:** 10.3389/fendo.2022.1028031

**Published:** 2022-11-10

**Authors:** Bo Wang, Chunhua Yang, Chuqiao Zhou, Shipeng Xiao, Hui Li

**Affiliations:** ^1^ Department of Orthopaedics, The First Hospital of Changsha, Changsha, Hunan, China; ^2^ Department of Orthopaedics, The Second Xiangya Hospital of Central South University, Changsha, Hunan, China; ^3^ Department of Spine Surgery, The Second Xiangya Hospital, Central South University, Changsha, Hunan, China

**Keywords:** osteosarcoma, non-coding RNAs, bibliometric analysis, VOSviewer, Citespace

## Abstract

**Background:**

Osteosarcoma is a common bone sarcoma that occurs in childhood and adolescence. Although research on non-coding RNAs (ncRNAs) of osteosarcoma has been developed rapidly in recent years, a specific bibliometric analysis on this topic has not yet been performed. The bibliometric analysis aims to summarize knowledge atlas, research hotspots, and emerging trends and to provide researchers with new perspectives in further studies.

**Methods:**

All publications regarding ncRNAs of osteosarcoma published from 2000 to 2021 were retrieved from the Web of Science Core Collection. Quantitative indicators including the number of publications and citations, H-index, and journal citation reports were analyzed by using Excel 2019 and R software. VOSviewer and CiteSpace were used to analyze the cooperation among countries/institutions/journals/authors and the co-occurrence of keywords, keywords bursts, and references.

**Results:**

A total of 3206 publications were extracted. A significant growth trend in the annual number of publications over the past 22 years is revealed (*R* ^2^ = 0.999). The most prolific country and institution were China (2260) and Shanghai Jiao Tong University (134), respectively. Professors Wang W and Liu W contributed the most to this field. The keywords were stratified into six clusters: Cluster 1 (apoptosis and growth), Cluster 2 (cancer and progression), Cluster 3 (microRNAs and downregulation), Cluster 4 (genes and differentiation), Cluster 5 (expression and biological functions), and Cluster 6 (metastasis). The long non-coding RNAs and circular RNAs have been considered as an important research hotspot in the near future.

**Conclusion:**

This study offers a scientific perspective on ncRNAs of osteosarcoma and provides researchers with valuable information to understand the knowledge structure and to identify emerging trends in this field.

## Introduction

Osteosarcoma is the most common primary bone tumor in children and adolescents, which occurs most frequently in the metaphysis of long bones, especially in the distal femur, proximal tibia, and humerus (1). There are approximately 4.4 cases of osteosarcoma per million children reported annually. Moreover, because of recurrence and metastasis, a poor prognosis means a high fatality rate (2, 3). Although comprehensive treatments including surgery, radiotherapy, and neoadjuvant and adjuvant chemotherapy have improved continuously in recent years, the 5-year survival rate of patients with osteosarcoma has no significant advances (4, 5). Unfortunately, a few patients with clinical metastasis at presentation do not survive for longer than 5 years (6, 7). Thus, it is important to seek new therapeutic methods for patients with osteosarcoma.

Osteosarcoma is derived from bone-forming mesenchymal cells with aberrant activation of oncogenes and inactivation of tumor suppressor genes (8–11). Apart from alterations of protein-coding genes, dysregulation of non-coding RNAs (ncRNAs) plays an important role in the regulation of several biological processes of osteosarcoma, including microRNAs (miRNAs), long non-coding RNAs (lncRNAs), small interfering RNAs (siRNAs), piwi-interacting RNAs (piRNAs), and circular RNAs (circRNAs) (12–16). For example, circRNAs are particular single-stranded RNA molecules with closed loops created by nonlinear backsplicing between a splice donor and an upstream splice acceptor. CircRNAs have been confirmed to play crucial roles in the regulatory element of the genome, including sponges of miRNAs and direct interactions with RNA-binding proteins (17–19). In addition, many specific miRNAs deregulated in osteosarcoma tumors can be demonstrated to be involved in cell differentiation, proliferation, and metastasis (20, 21). The roles and mechanisms of non-coding RNAs in osteosarcoma are not fully elucidated; however, studies on them have been increasing rapidly and gaining increased attention.

In this study, bibliometrics can be used for the quantitative analysis of hotspots in the literature. The main objectives of this study were to summarize the current status of and reveal future developing trends in the knowledge domain of ncRNAs on osteosarcoma. It is more crucial to propose research frontiers and potential hotspots in the near future.

## Materials and methods

### Source of bibliometric data and search strategies

Literature data on ncRNAs of osteosarcoma were identified and downloaded from the Science Citation Index Expanded (SCI-Expanded) of the Web of Science Core Collection (WoSCC) database. Medical Subject Heading (MeSH) was applied in the search. Language was limited to English; literature types were limited to original article and review. The timespan for data retrieval was 2000 to 2021.The scientific literature was searched with the following search strategy: (osteosarcoma OR ‘osteogenic sarcoma’) AND (‘long non-coding RNA’ OR ‘lncRNA’ OR ‘long ncRNA’ OR ‘ncRNA’ OR ‘non-coding RNA’ OR ‘miRNA’ OR ‘microRNA’ OR ‘miRNAs’ OR ‘circRNA’ OR ‘circular RNA’ OR ‘ceRNA’ OR ‘competing endogenous RNA’ OR ‘piRNA’ OR ‘Piwi-interacting RNA’). All data utilized in this study were downloaded from known databases, and no ethical approval was required.

### Data export and extraction

**Figure 1:**
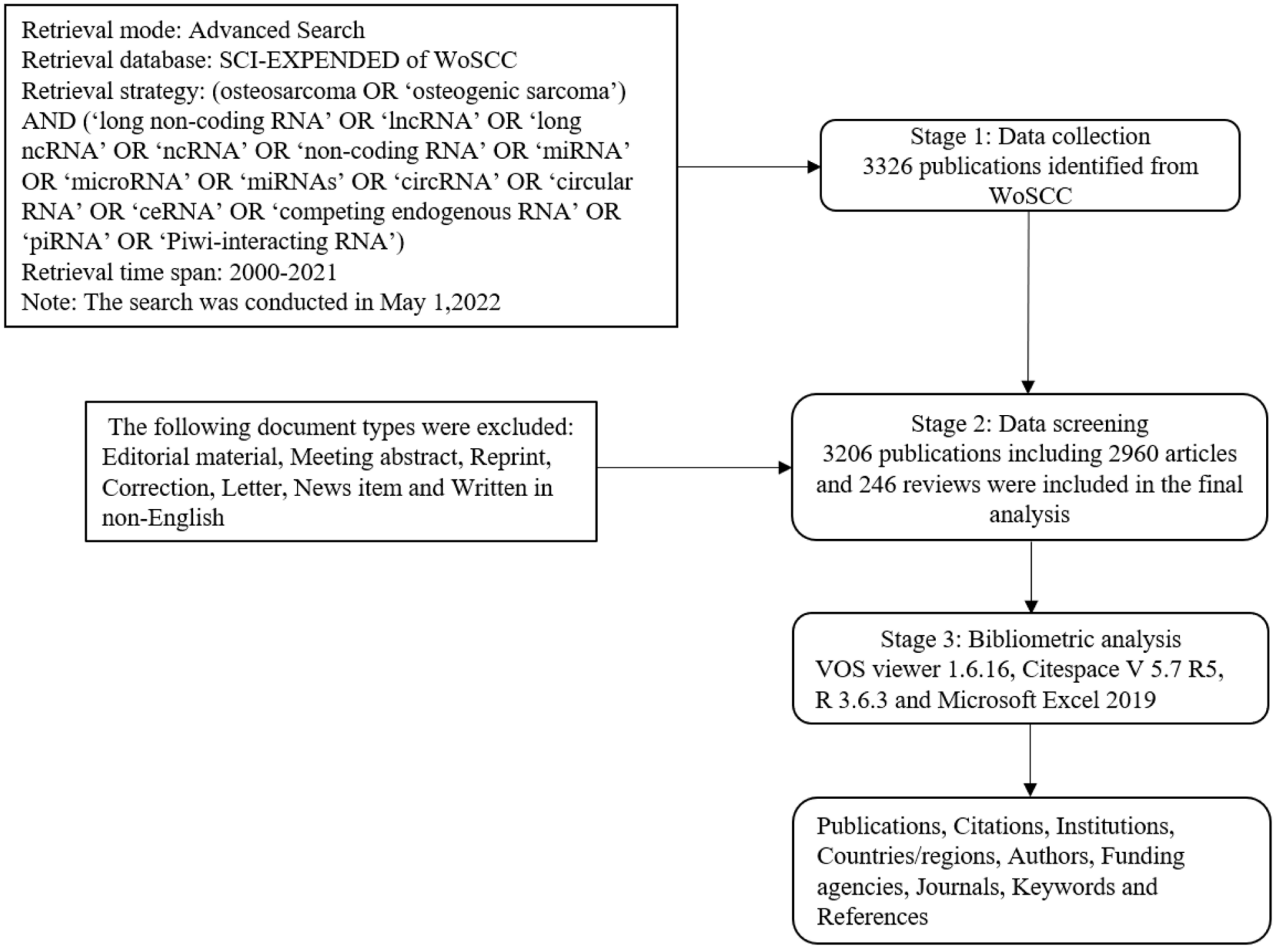
Flow diagram of the literature search and selection process.

The process of data searches was conducted on a separate day and by two authors independently. All collected data were saved as 'plain text' with the basic information by using the function of 'export' in WoSCC; the basic information in each publication was collected including published date, title, source journal, author, country/region, institution, funding sources, abstract, keywords, number of citations, and Hirsch index (H-index). The Hirsch index (H-index) is used to quantify an individual’s scientific research output and measure his or her citation impact (22).The above process was conducted by two authors to ensure authenticity and avoid duplication, and any disagreements were solved through discussion, if necessary, with the third author. Moreover, journal impact factors (JIFs) were collected from the 2022 Journal Citation Reports. The detailed literature search and selection process are shown in **Figure 1**.

### Bibliometric and statistical analysis

To obtain a more comprehensive analysis, three bibliometric tools, including an R software package ('bibliometrix') and two software packages, were used to conduct this research. Firstly, the bibliometrix package in R 4.0.3 was used to convert and analyze automatically the bibliographic information of the included publications and included the distribution of countries/regions, years of publication, and source journals. Furthermore, academic cooperation networks between countries/regions were conducted. Then, VOSviewer 1.6.16 and CiteSpace 5.7.R5 were further used for mapping and visualization of the bibliometric networks of included publications. VOSviewer is one of the frequently and freely available bibliometric tools for constructing and viewing bibliometric maps (23). In this research, VOSviewer was used to construct and visualize the following network maps: network map of co-citation authors and journals, and co-occurrence analysis of keywords. Based on the frequencies of items where they occur together, co-citation and co-occurrence analysis mean that each item was connected with others by links, which could reflect the relationships of items (24). Meanwhile, in the network visualization, each node represents a different item, such as countries/regions, journals, or keywords, and the different colors of nodes indicated different taxonomies or occurrence frequencies. The sizes of nodes represent the number of citations or occurrences, with bigger nodes representing a higher level of citations or occurrences (25). The links between the nodes reflect the correlation between co-citations or co-occurrences of items, and the thickness of the links represents the strength of the links; thus, total link strength (TLS) is used to quantitatively evaluate the links (23, 25). A detailed description of the maps can be found in the software manual (https://www.vosviewer.com/documentation).

In addition to VOSviewer software, we also used another bibliometric software, called CiteSpace, which is a Java-based scientometrics research software package that is used to analyze and visualize the hot spots and research frontiers in the scientific literature of a discipline or knowledge domain in a certain period (26, 27). In the present research, CiteSpace was applied to conduct and visualize the research cooperation relationship of authors and institutions; the timeline view map of co-citation references; and references with the strongest citation bursts. All visualization maps constructed by CiteSpace also comprised nodes and links representing different items similar to those of VOSviewer. CiteSpace is able to construct some distinctive types of visualization maps, such as the cluster view map and the timeline view map. For example, the timeline view map, based on an important function of time-slicing, provides information on some research areas within corresponding time periods by mapping the highly cited and pivotal documents (27). In CiteSpace, betweenness centrality (BC) is a key indicator that could identify the relative importance of a node within the networks, and nodes with the highest BC value (≥0.1) are known as hub nodes that are marked with purple rings (28). A more detailed description of the software can be found in the operational manual (http://cluster.ischool.drexel.edu/cchen/citespace/CiteSpaceManual).

R software (version 4.03) and Microsoft Excel 2019 were used for descriptive statistical analysis, data fitting, and plotting graphs of publications including the number of publications per year, original countries/regions, authors, journals, institutions, H-index, and funding agencies. The growth rate of publications over time was computed according to the previously described calculation formula (29).

## Results

### Publication outputs and trends

**Figure 2:**
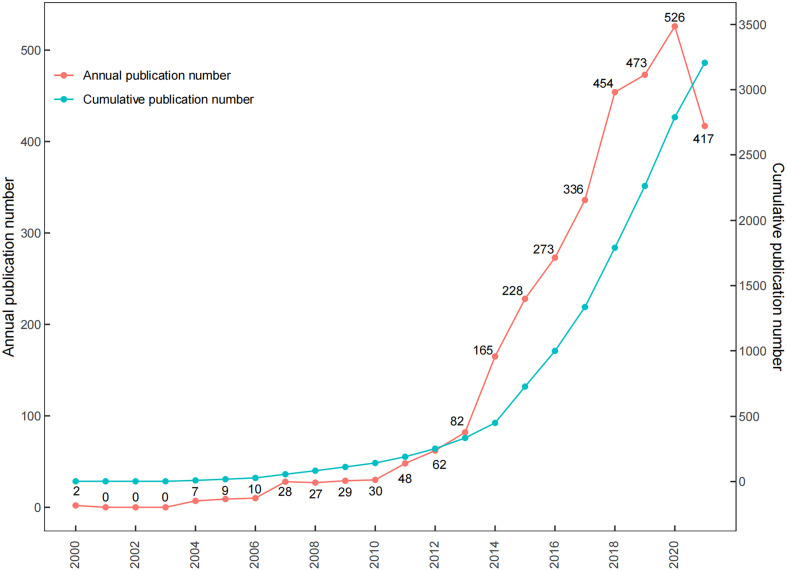
The numbers of annual and cumulative publications.

After removing 120 unqualified publications, a total of 3206 publications obtained from the WoSCC including 2960 articles and 246 reviews met the inclusion criteria from 2000 to 2021. **Figure 2** presents the specific numbers of annual publications about ncRNAs of osteosarcoma. Moreover, the cumulative publication number rose rapidly in the recent years. The model fitting curve revealed a significant growth trend in the annual number of publications over the past 21 years (*y=*1.147×10^-9^*x ^4^ – 9.259*x*,*R* ^2^ = 0.999). From 2000 to 2021, the average growth rate of publications was 33.25%.

### Analysis of countries/regions and funding agencies

**Figure 3:**
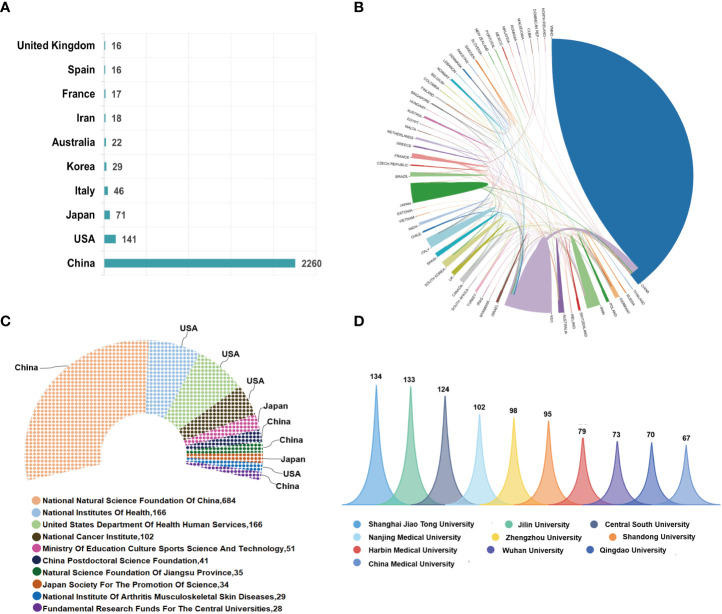
**(A)** The total number of publications in the top 10 prolific countries from 2000 to 2021. **(B)** International collaboration of countries/regions in this domain. The thickness of the line reflects the frequency of the cooperation. The thicker the line, the stronger the cooperation. **(C)** The top 10 most active funding agencies in this field. **(D)** The top 10 most productive institutions in this field.

Most of the 3206 publications concerning ncRNAs of osteosarcoma were distributed among 38 active countries/regions, among which the top 10 countries in terms of literature volume accounted for 2636 (82.22%) publications. Specifically, China had published the most publications with 2260 (70.49%) articles/reviews, followed by the USA (141, 4.4%), Japan (71, 2.21%), Italy (46, 1.43%), Korea (29, 0.9%), Australia (22, 0.69%), Iran (18, 0.56%), France (17, 0.53%), Spain (16, 0.5%), and the United Kingdom (16, 0.5%); the abovementioned is shown in **Figure 3A**. International collaboration of countries in this field was also analyzed and is further demonstrated in **Figure 3B**. Extensive collaboration was carried out between active countries. For example, China collaborated most closely with the USA, Japan, and the United Kingdom. More active cooperation was demonstrated among the three countries—the USA, Japan, and the United Kingdom—as well. **Figure 3C** demonstrates the world’s top 10 most active funding agencies that sponsored the research about ncRNAs of osteosarcoma. Among them, four were from China, four were from the USA, and the remaining two agencies were from Japan. The National Natural Science Foundation of China (NSFC) ranked first, supporting the highest number of 684 studies, followed by the National Institutes of Health (NIH) and the United States Department of Health and Human Services (HHS), both of which were from the USA and supported 166 studies.

### Analysis of the most prolific institutions and influential authors

**Figure 4:**
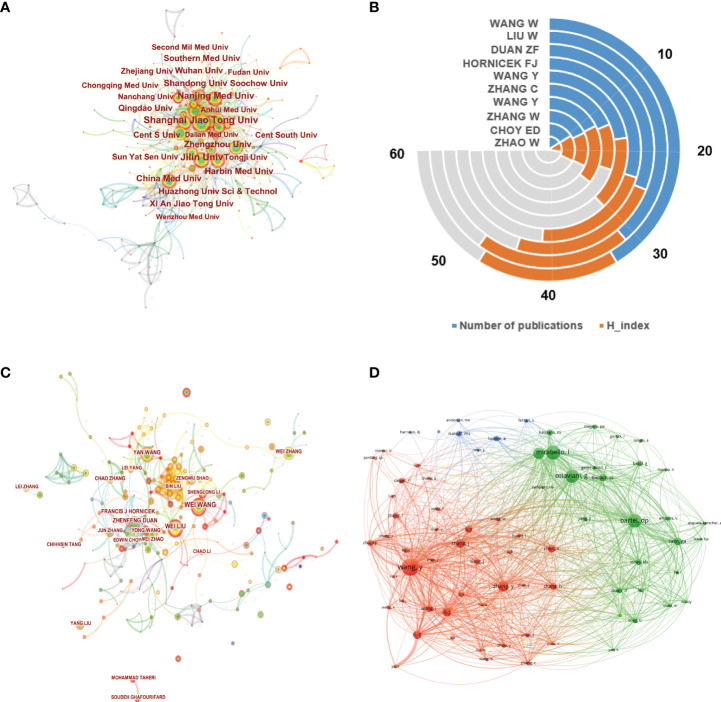
**(A)** Network map of institution co-authorship analysis based on CiteSpace. In the visualization map, each node represents an institution, and its size is proportional to the number of publications. The connecting line between nodes represents the strength of the cooperation relationship. **(B)** The total number of publications and H-index of top 10 productive authors in this field. **(C)** The cooperation network map of productive authors generated by CiteSpace. The graphical explanations are the same as in A. **(D)** Author co-citation analysis by VOSviewer. Each node represents a different author, and node size indicates the quantity of citations. The connection between the nodes represents a citation relationship, and the thickness of the lines indicates citation strength.

The top 10 institutions involved in this field are demonstrated in **Figure 3D**. Shanghai Jiao Tong University was the largest contributor with 134 publications, followed by Jilin University and Central South University, with 133 and 124 publications, respectively. The network visualization map of cooperation relationships between institutions is illustrated in **Figure 4A**. However, all of the institutions had a BC value of less than 0.1, indicating that none of the institutions occupied the central position in the cooperation network. Only institutions with a minimum of 30 publications were included in the network map. **Figure 4B** demonstrates the top 10 authors who published the greatest number of papers. Besides Hornicek FJ, Choy ED, and Duan ZF, the other authors come from China. Wang W from China contributed the maximum number of publications (30), followed by Liu W with 31 papers and Duan ZF with 25 papers. **Figure 4C** illustrates the author cooperation network map of the most productive authors. Both Wang W and Liu W contributed not only a lot; the other authors had also more collaborations with them. In addition, the co-citation network among authors was conducted using VOSviewer. As displayed in **Figure 4D**, only those authors with a minimum of 100 citations were included. There were 74 nodes, 3 clusters, and 2663 links in the network map. Among them, Wang Y had the largest number of citations (557 times) and occupied the maximum node with TLS.

### Analysis of the higher−impact journals

**Figure 5:**
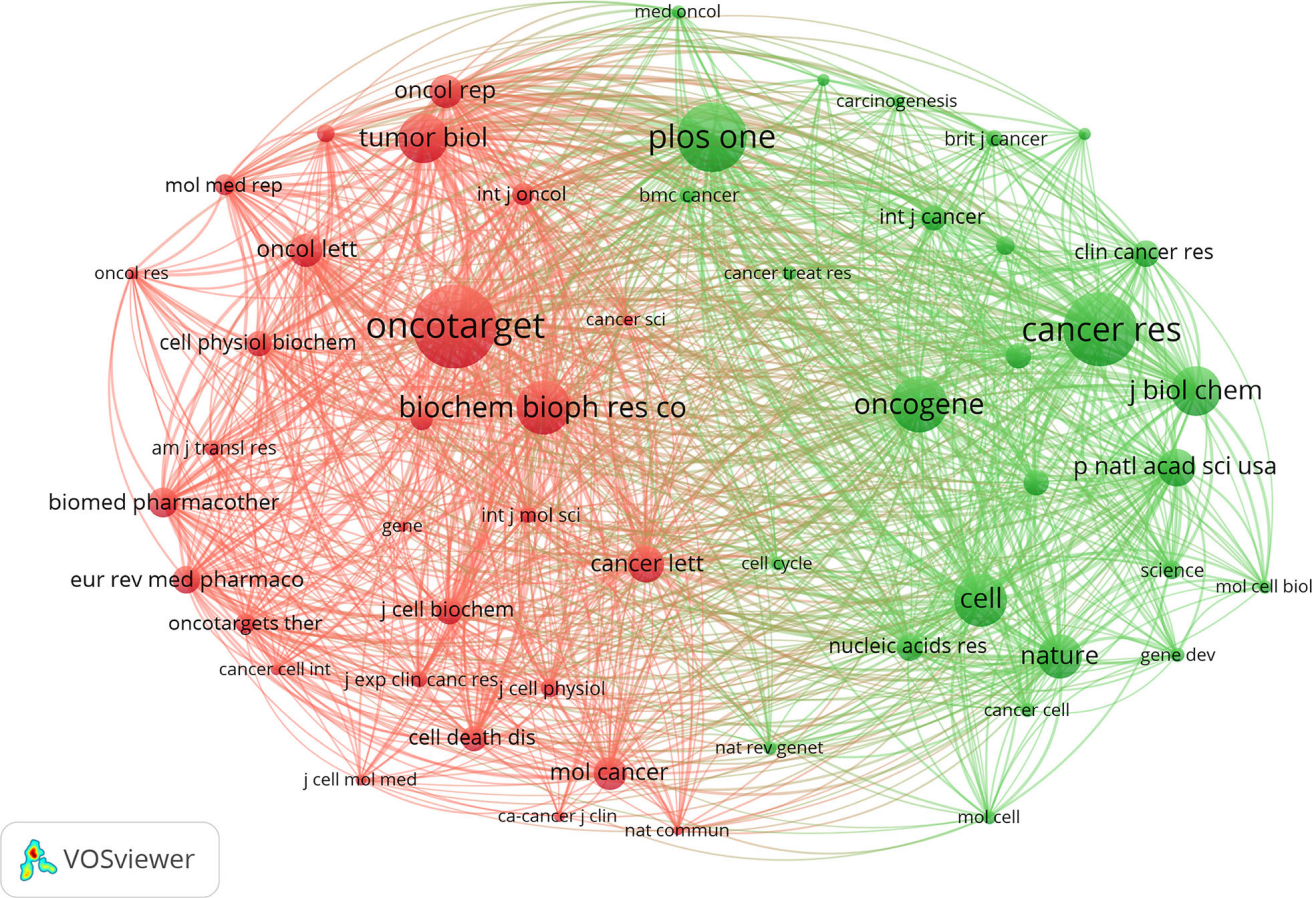
The network visualization map of journal co-citation analysis by VOSviewer. The explanation of the nodes and links in the map is identical to that described in **Figure 4D**.

In total, 4879 journals were involved in this research field. The top 10 most prolific journals are listed in **Table 1**. *Oncology Letters* (JIF 3.111) has published the greatest number with 120 papers, accounting for 3.74% of the publications, followed by *Molecular Medicine Reports* (JIF 3.423) and *European Review for Medical and Pharmacological Sciences* (JIF 3.784) with 99 and 93 papers, respectively. According to the JCR 2022 standards, the top 10 most active journals were classified as Q1 in 1, Q2 in 2, and Q3 in 4. **Figure 5** shows the network visualization map of journal co-citation analysis. Only journals with more than 500 citations were displayed. There were 55 nodes, 2 clusters, and 1485 links in the network map. Of the 55 journals shown on the map, the top five co-cited journals were *Oncotarget, PLOS One, Cancer Research, Tumor Biology*, and *Biochemical and Biophysical Research Communications*, which possessed the top five maximum nodes with TLS.

**Table 1:** Top 10 journals with most publications about ncRNAs of osteosarcoma.

Ranking	Journal title	Country	Output	% of 3206	H-index	JIF(2022)	Quartile in category (2022)
1	*Oncology Letters*	Greece	120	3.74	19	3.111	Q3
2	*Molecular Medicine Reports*	Greece	99	3.09	14	3.423	Q3
3	*European Review for Medical and Pharmacological Sciences*	Italy	93	2.90	18	3.784	Q2
4	*Oncotarget*	United Kingdom	80	2.50	24	NA	NA
5	*Tumor Biology*	USA	77	2.40	8	NA	NA
6	*Biochemical and Biophysical Research Communications*	USA	68	2.12	25	3.322	Q3
7	*Oncology Reports*	Greece	67	2.09	13	4.136	Q3
8	*Biomedicine & Pharmacotherapy*	France	63	1.97	24	7.419	Q1
9	*International Journal of Clinical and Experimental Pathology*	USA	59	1.84	5	NA	NA
10	*Oncotargets and Therapy*	United Kingdom	56	1.75	16	4.345	Q2

NA, Not available.

### Analysis of highly cited references

**Figure 6:**
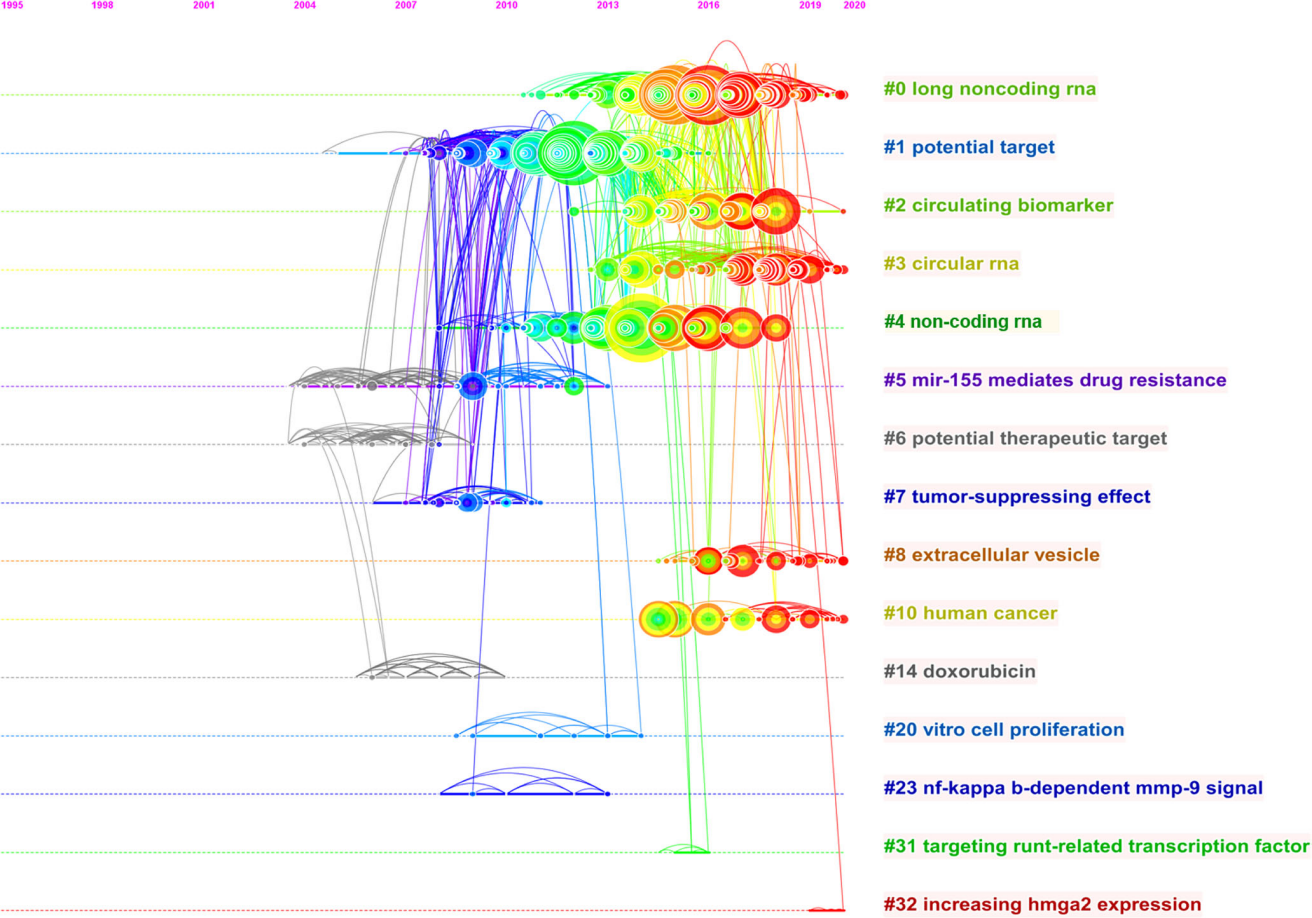
The timeline view map of reference co-citation analysis. For each cluster, the position of each node shows the time of publication of the document, and the node size represents the number of citations.

**Figure 7:**
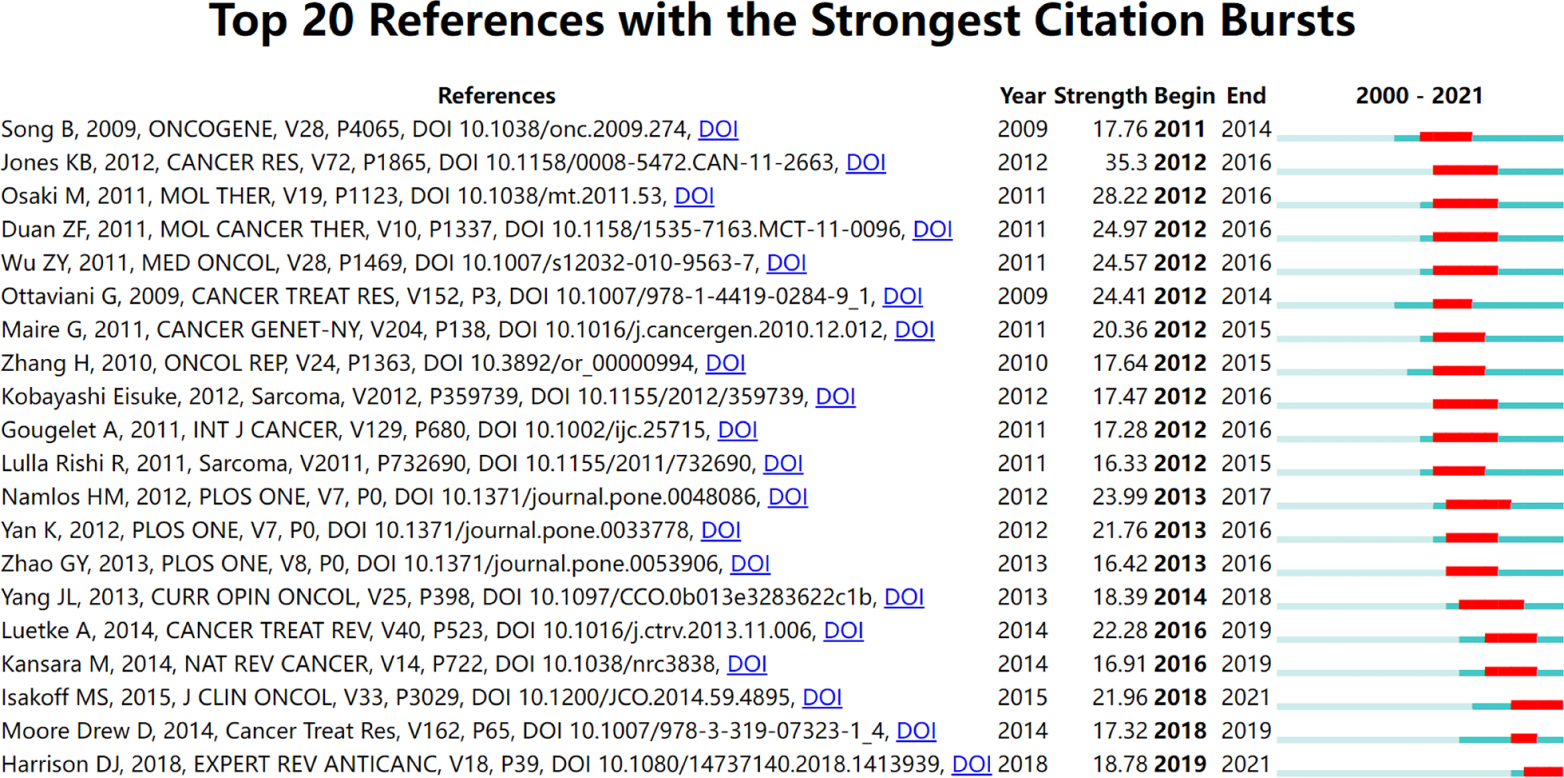
The top 20 references with the strongest citation burst from 2000 to 2018.

The reference co-citation relationship was visualized in a co-citation network. In general, the weighted average silhouette value (S value) and the modularity value (Q value) are two indicators to evaluate the significance of clustering, and if the S value is over 0.7 and the Q value is over 0.3, the cluster is efficient and convincing. In this study, the mean value of S equals 0.9204 and that of Q equals 0.7567, indicating the rationality of this clustering strategy. As shown in **Table 2** and **Figure 6**, all the nodes in the reference co-citation network map could be grouped into 15 major clusters (also the silhouette value of nearly all clusters exceeded over 0.9), which were produced by the log-likelihood ratio. The top five clusters were mainly around long non-coding RNA, potential target, circulating biomarker, circular RNA, and non-coding RNA. Moreover, as shown in **Figure 7**, the top 20 references with the strongest citation bursts were identified in terms of their burst values. These highly cited papers were published between 2009 and 2018. The most highly cited paper was written by Jones KB et al. (31), followed by Osaki M et al. (2011) (32) and Duan ZF et al. (2011) (33).

**Table 2:** Main clusters of co-cited references.

Cluster ID	Size	Silhouette	Label (LLR)	Mean(Year)
#0	217	0.914	Long non-coding RNA	2016
#1	196	0.835	Potential target	2011
#2	107	0.887	Circulating biomarker	2016
#3	102	0.977	Circular RNA	2017
#4	65	0.949	Non-coding RNA	2013
#5	49	0.97	MiRNA-155 mediates drug resistance	2008
#6	37	0.962	Potential therapeutic target	2006
#7	30	0.956	Tumor-suppressing effect	2009
#8	29	0.986	Extracellular vesicle	2017
#10	15	0.999	Human cancer	2018
#14	13	0.966	Doxorubicin	2008
#20	8	0.997	Vitro cell proliferation	2011
#23	6	0.996	NF-kappa B-dependent MMP-9 signal	2010
#31	4	0.991	Targeting Runt-related transcription factor	2015
#32	4	1	Increasing HMGA2 expression	2019

### Analysis of the most concerned keywords

**Figure 8:**
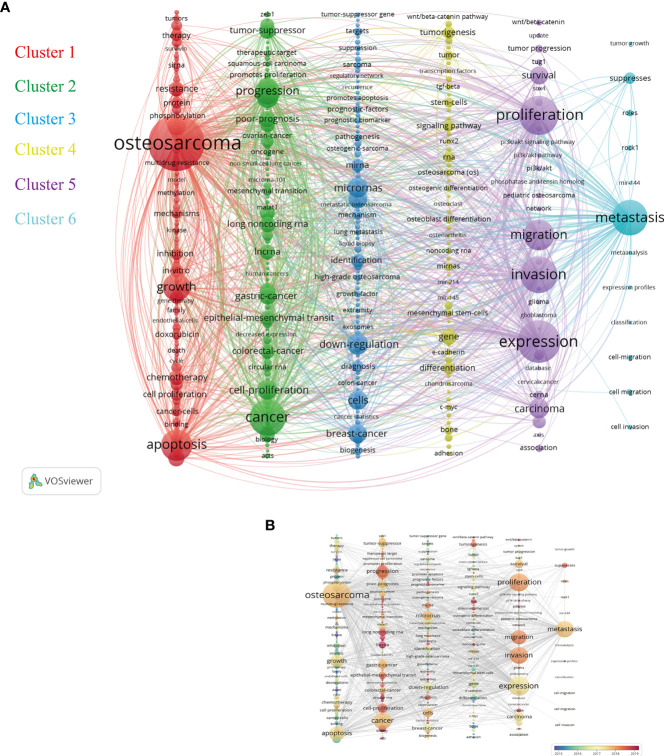
**(A)** Network visualization map of the keyword co-occurrence analysis by VOSviewer. Keywords with close relationship will be assigned to one cluster with the same color. The graphical explanations are the same as in Figure 4D. **(B)** Overlay visualization map of keyword analysis in this field. The color of each node shows the average appearing year of the keyword.

The keyword co-occurrence analysis largely determined developing trends, related hot topics, and tracing scientific development. Only keywords with a minimum of 10 occurrences were included in the analysis. Of all 8084 keywords, 396 keywords met the criterion. Based on the research categories of these keywords, VOSviewer software divided these keywords into several major clusters with different colors. As illustrated in the network visualization map of **Figure 8A**, the total six highly relevant clusters were identified and represented the topical issues in ncRNAs of osteosarcoma research, including Cluster 1 (apoptosis and growth), Cluster 2 (cancer and progression), Cluster 3 (microRNAs and downregulation), Cluster 4 (genes and differentiation), Cluster 5 (expression and biological functions), and Cluster 6 (metastasis). The primary keywords of these clusters were osteosarcoma, cancer, downregulation, gene, expression, and metastasis. Osteosarcoma and expression were the most frequent keywords, followed by proliferation and invasion. Moreover, these above keywords were consistent with our research theme. In addition, the overlay visualization map of keyword co-occurrence analysis is provided in **Figure 8B**.

## Discussion

### Research tendency

Our study examined the patterns of research on ncRNAs of osteosarcoma by using bibliometric and visualized analysis. Through the formula of model fitting curve in this study, the number of studies is demonstrating exponential growth and undergoing tremendous expansion in this field. The number of scientific outputs in this field has been increased from 2 in 2000 to 417 in 2021, and 3016 (94.07%) of the total publications were published in the last 10 years. The increasing trend was similar to the increasing global incidence of osteosarcoma in recent years (30, 34). It was predicted that there is apparently a brighter perspective for research on ncRNAs of osteosarcoma in the coming years.

### Knowledge atlas of countries/regions, institutions, and funding agencies

Among the 53 countries/regions that participated in the publication of studies in this domain, China was the most productive country, which also published the most articles in cooperation with many countries and was far ahead of the second-ranked USA. These results indicated that China might have a significant impact on the research in this field and possess the strongest collaborations worldwide. In terms of research institutions, a total of 2311 institutions contributed to the publications on ncRNAs of osteosarcoma; however, all the top 10 most influential institutions were from China. Notoriously, the research and development on ncRNAs of osteosarcoma required a large amount of both human and financial resources, which reflected the comprehensive strength of a country. It is proven again that China had made considerable progress in this field despite being a developing country. Among the top 10 funding agencies, China, the USA, and Japan divided up themselves and, meanwhile, the economic aggregates of the three countries are among the top three in the world.

### Influential authors and journals

The H-index is an important indicator to quantify and standardize the scientific output and academic status of a researcher, which combines a researcher’s total number of publications and number of citations (24). Researchers with similar H-indices should be equivalent in terms of scientific impact in a specific field, which is helpful for researchers to learn trends of productive authors and seek potential cooperative opportunities with them. In this study, Wang W, Liu W, and Duan ZF were the top three contributors in this field. Wang W and his team mainly focused on understanding the targeted regulatory networks of non-coding RNAs in osteosarcoma (35). Duan ZF was the leading author in the H-index, which was from the USA and mainly focused on basic studies on osteosarcoma, especially emerging biomarkers and their clinical implication in osteosarcoma (36), which facilitated the diagnosis of osteosarcoma, the evaluation of treatment effects, and the prediction of prognosis. The co-citation analysis was usually used to evaluate the academic influence of a journal or a researcher (37). As shown in **Figure 4D**, Wang Y, Bartel DP, and Luetke A had respectively occupied the maximum nodes with the largest citations in these three corresponding clusters. As for journal analysis, *Biochemical and Biophysical Research Communications* had the topmost H-index, followed by *Oncotarget* and *Biomedicine & Pharmacotherapy*. The top 10 journals with the most publications were affiliated with the USA and some Western European countries. The Chinese had played a significant role in the advancement of biological processes and scientific research on ncRNAs of osteosarcoma. China had no top international journals in this field yet.

### Research focuses and frontiers: reference and keyword analysis

Reference co-citation analysis was also an important parameter analysis to assess the research focuses in a certain field. In this study, the total 15 major clusters and their corresponding labels were demonstrated in the co-citation network map. As shown in **Figure 6**, the top five largest labels revealed that some newly discovered non-coding RNAs had become the research focus in recent years. The ‘long non-coding RNA’ was the largest one of these corresponding labels of clusters, which indicated that it might be an important member of the information regulatory network in osteosarcoma cells and thus more extensively researched in the next few years (38, 39). The ‘circular RNAs’ had become a newly rising research hotspot and played a vital role in the functions and mechanisms of action in osteosarcoma (40). **Figure 7** shows the top 20 strongest burst references. A study by Jones KB et al. (31) represented the strongest burst by the end of 2016. This research revealed the functions of some miRNAs and related highlighting pathways in osteosarcoma, and then established an miRNA signature associated with the pathogenesis of osteosarcoma as well as critical pretreatment biomarkers, followed by Osaki M et al. (32) who represented the second strongest burst by the end of 2016. This research demonstrated that the correlation between the downregulation of miR-143 and the lung metastasis of human osteosarcoma and, further, systemic injection of miRNA/atelocollagen complexes might have the therapeutic potential for the prevention of lung metastasis from osteosarcoma. The following two studies by Duan ZF et al. (33) and Wu ZF et al. (41) focused on the biological function of miR-199a-3p and miR-21 in human osteosarcoma cells, respectively, which might provide potential therapeutic benefits in osteosarcoma. These studies, which were involved in some miRNAs, had gained considerable attention in the past few years and suggested that these classic miRNAs might play a key role in regulating cellular processes in osteosarcoma and need to be further extensively studied.

Keyword co-occurrence analysis was a significant bibliometric method that could provide the knowledge network map and the indicator of frontier topics. In addition to this, VOSviewer software can assign different colors to all the included keywords based on their average year of appearance. It can be seen that most of the research studies prior to 2019 mainly focused on the biological function of osteosarcoma (including invasion, migration, and proliferation) and its metastasis. However, except for some known miRNAs, long non-coding RNAs and circular RNAs gradually gained attention afterward.

Cluster 1 consisted of 134 keywords, mainly focused on the apoptosis and growth of osteosarcoma and especially therapy. Most chemotherapy drugs can cause cell apoptosis and death but with some bad complications and severely fatal toxicity (42). In addition, multidrug resistance frequently leads to the failure of chemotherapy for osteosarcoma (43). Most of the keywords in Cluster 1 had emerged earlier than other clusters and had been related to these in Cluster 5. Clusters 2 and 3 mainly were related to other metastatic cancers, progression, prognosis, and newly discovered non-coding RNAs. Osteosarcoma could nearly metastasize to any parts or organs, mostly to the lungs, which indicated poor prognosis in osteosarcoma patients^44^. Moreover, some ncRNAs were associated with both osteosarcoma and digestive system tumors (44–46). Cluster 4 majorly focused on osteosarcoma cell gene and differentiation. Osteosarcoma tumor stem cell–related genes and their signaling pathways had acted a vital role in the development of osteosarcoma and cell differentiations (47, 48), which had been considered to be promising therapeutic targets for osteosarcoma and had been extensively studied in the past years. Cluster 5 was mainly about osteosarcoma biological behavior studies, including the invasion, migration, and proliferation of osteosarcoma, which were the basic and crucial research objectives. The PI3K/Akt signaling pathway took part in the above biological behavior and was regulated by upstream ncRNAs in osteosarcoma cells, for example, circular RNAs and miRNAs (49, 50). As for Cluster 6, the frequently used keyword was metastasis, which was associated with biological functions of many non-coding RNAs, including miRNA-27a (51), long non-coding RNA metastasis-associated lung adenocarcinoma transcript 1 (MALAT1) (52), and circular RNA circ_HIPK3 (53). In terms of MALAT1, a study suggested that high MALAT1 expression is also associated with advanced clinicopathological features as well as poor prognosis in osteosarcoma (54), which might emerge as potential therapeutic targets in the treatment of patients with osteosarcoma. Most long non-coding RNAs could play regulatory roles in various biological processes of osteosarcoma cells through some miRNA-related axes, such as lncRNA HOTAIR (55) and lncRNA HCP5 (56). Evolutionary trends of the keywords in ncRNAs of osteosarcoma indicated that non-coding RNAs were characterized by the lack of ability to encode proteins; however, they gradually attracted great attention and became the latest research hotspots in recent years.

### Strengths and limitations

To our knowledge, this is the first-ever study to perform bibliometric analysis about non-coding RNAs of osteosarcoma. To get more comprehensive and reliable analysis results, four visualization software tools were used to assess the latest research progress and identify the hotspots in this field. However, in spite of the strengths mentioned above, several limitations should be noted. Firstly, only data from the WoSCC database were analyzed, excluding other databases such as PubMed, Scopus, and Embase. Secondly, only original research articles and reviews were included, and only English-language studies were included. Thirdly, articles published in 2022 were not included in this analysis; it is unavoidable that several latest publications were omitted.

## Conclusion

Summarizing these findings, the ascending trend in the annual number of publications indicates that ncRNAs of osteosarcoma have attracted researchers’ great interest, especially in the last 10 years. China is a major contributor in this field. The most productive institution was Shanghai Jiao Tong University. Professor Wang W was the most influential author with the highest number of publications. According to keyword analysis, it is recommended to pay attention to potential research hotspots, such as lncRNAs, circRNAs, and ceRNAs. In general, these findings could provide several promising directions for future research and also might open up new therapeutic perspectives.

## Data availability statement

The raw data supporting the conclusions of this article will be made available by the authors, without undue reservation.

## Author contributions

WB and YC contributed to the conception of the study. WB and XS performed the experiment and contributed significantly to the analysis and manuscript preparation. WB, ZC and LH performed the data analyses and helped write the manuscript. YC and LH helped perform the analysis with constructive discussions. All authors contributed to the article and approved the submitted version.

## Funding

This study was supported by the Research Project of the Hunan Health Commission (grant number 202204073071).

## Conflict of interest

The authors declare that the research was conducted in the absence of any commercial or financial relationships that could be construed as a potential conflict of interest.

## Publisher’s note

All claims expressed in this article are solely those of the authors and do not necessarily represent those of their affiliated organizations, or those of the publisher, the editors and the reviewers. Any product that may be evaluated in this article, or claim that may be made by its manufacturer, is not guaranteed or endorsed by the publisher.
